# Induction Chemotherapy in Head and Neck Squamous Cell Carcinoma: A Question of Belief

**DOI:** 10.3390/cancers11010015

**Published:** 2018-12-22

**Authors:** Andy Karabajakian, Max Gau, Thibault Reverdy, Eve-Marie Neidhardt, Jérôme Fayette

**Affiliations:** Rhône-Alpes, Centre de Lutte Contre le Cancer Léon Bérard, 69008 Lyon, France; andy.karabajakian@gmail.com (A.K.); Max.gau@lyon.unicancer.fr (M.G.); Thibault.Reverdy@lyon.unicancer.fr (T.R.); eve.marie.neidhardt@lyon.unicancer.fr (E.-M.N.)

**Keywords:** induction, larynx preservation, controversial, inconclusive, de-escalation, toxicity, high-risk

## Abstract

Induction chemotherapy (IC) in locally advanced head and neck squamous cell carcinoma (LA HNSCC) has been used for decades. However, its role is yet to be clearly defined outside of larynx preservation. Patients with high risk of distant failure might potentially benefit from sequential treatment. It is now widely accepted that TPF (docetaxel, cisplatin, and fluorouracil) is the standard IC regimen. Essays that have compared this approach with the standard of care, concurrent chemoradiotherapy (CCRT), are mostly inconclusive. Radiotherapy (RT) can be used in the post-IC setting and be sensitized by chemotherapy or cetuximab. Again, no consensus exists but there seems to be trend in favor of potentiation by cisplatin. Less toxic schemes of IC are tested as toxicity is a major issue with TPF. IC might have an interesting role in human papilloma virus (HPV)-related LA HNSCC and lead to CCRT de-escalation.

## 1. Introduction

Every year, more than half a million new head and neck cancers are diagnosed worldwide, with 380,000 deaths [1], making these cancers responsible for around 3% of malignancies in the U.S.A. [2] and 4% in Europe [3], mostly in male subjects.

Squamous cell carcinomas of the oral cavity, the pharynx, and the larynx (the most frequent) are linked to smoking and alcohol consumption and are different from squamous cell carcinoma of the oropharynx (OPC) caused by human papilloma virus (HPV), especially for young or non-smoker patients. HPV-induced cancers and toxic-use related cancers are described in the 2017 WHO classification and are considered as two different clinical entities, with different oncogenic activation pathways and prognostics [4]. The incidence of OPC is rising, especially amongst men [5].

Locally advanced head and neck squamous cell carcinoma (LA HNSCC) can be classified as resectable, unresectable, or of a borderline category, with a poor functional outcome (e.g., total glossectomy) [6]. This last category of patients could benefit from an aggressive non-surgical approach instead of surgery [6].

When the disease is operable and the main objective of treatment is organ preservation, CCRT or the sequential approach with TPF (docetaxel, cisplatin, and fluorouracil) followed by radiotherapy (with or without a sensitizing agent) are the two options. Outside of organ preservation for resectable disease and also in the case of inoperable disease, concurrent chemoradiotherapy (CCRT; radiotherapy and cisplatin, carboplatin and fluorouracil or cetuximab (for cisplatin-unfit patients)) is the current standard of care. The use of induction chemotherapy (IC) followed by radiotherapy or CCRT in this later setting is controversial. 

In this paper, we will review the use of IC in HNSCC and how TPF came to be the standard, its role in larynx preservation, and its potential use for high risk patients and for treatment de-escalation. We will also discuss how different studies compared IC with CCRT and the controversies that surround them.

## 2. Induction Chemotherapy and How TPF Came to be 

The MACH-NC meta-analysis of 93 trials and 17,346 patients does not show a significant benefit for IC [7]. However, a significant improvement in overall survival (OS) appears when only cisplatin and 5-FU based IC is considered in comparison with radiotherapy (RT) [7]. 

In a randomized phase III study of 237 patients with inoperable stage III or IV HNSCC, induction chemotherapy by four cycles of cisplatin (100 mg/m*2* q3w) and 5-FU (1000 mg/m^2^/days for 5 consecutive days q3w) (PF), followed by RT was compared with RT alone. OS at 5 and 10 years was 21% and 16% with induction compared with 8% and 6% without induction (*p* = 0.04) [8]. In this trial, induction chemotherapy was not compared with the standard CCRT scheme.

The association of docetaxel (75 mg/m^2^ as on day 1 q3w) with PF (TPF) has shown an additional benefit. Its superiority was confirmed in a meta-analysis by Blanchard et al. [9] of five randomized trials representing 1772 patients comparing PF induction chemotherapy with taxanes (docetaxel or paclitaxel), cisplatin, and fluorouracil (Tax-PF). The Hazard ratio (HR) of death was 0.79 (95% CI: 0.70–0.89; *p* < 0.001; absolute benefit at 5 years: 7.4%) in favor of Tax-PF. Patients who receive TPF versus PF experience benefits in progression-free survival (PFS), OS and in locoregional and distant failure rate. It is of note however that a potential bias might be the pooled analysis of five heterogeneous studies and the fact that responders and non-responders to IC received different sequential treatments. 

In a randomized phase 3 trial of 501 patients, the American TAX 324 study, induction with three cycles of TPF (75 mg/m^2^ docetaxel, 100 mg/m^2^ cisplatin, both on day 1 and 1000 mg/m^2^/day 5-FU by continuous intravenous (i.v.) infusion for four consecutive days), followed by CCRT (with weekly carboplatin) improved OS at 3 years from 48 to 62% compared with PF; the median OS was 71 versus 30 months (*p* = 0.006) [10]. Fewer patients had treatment delays in the TPF arm versus in the PF arm (29% versus 65%, respectively). Only grade 3 or 4 neutropenia was more frequent (83% vs. 56%) in terms of adverse events. Patients included had a disease of either unresectable or of low surgical curability, as well as patients with locally advanced head and neck squamous cell carcinoma (LA HNSCC) who were candidates for organ preservation strategy.

Four cycles of TPF (75 mg/m^2^ docetaxel, 75 mg/m^2^ cisplatin, both on day 1 and 750 mg/m^2^/day 5-FU by continuous i.v. infusion for five consecutive days) followed by RT alone also improved PFS versus PF in the TAX 323/EORTC 24,971 trial of 358 patients with previously untreated, unresectable LA HNSCC. (11.0 vs. 8.2 months; *p* = 0.007) [11]. It also prolonged OS (median, 18.8 months versus 14.5 months in the TPF versus PF arms, respectively). More patients completed treatment in the TPF arm than did patients in the PF arm (75.7% versus 65.7%, respectively), and fewer toxic deaths were encountered (2.3% versus 5.5%). Again, apart from alopecia and infections, only grade 3 or 4 neutropenia was more frequent.

Across the five trials in the aforementioned meta-analysis [9], patients who received TPF had fewer grade 3/4 mucositis and experienced lower frequencies of nausea, vomiting, stomatitis, and hearing loss. The only adverse events that appeared to be more prevalent with TPF versus PF were neutropenia, febrile neutropenia, and leukopenia.

In addition, significant improvement in quality of life (e.g., swallowing, coughing) in the TAX 323 trial was observed in the TPF arm vs PF arm [12], and economical analysis of both the TAX 323 and 324 studies showed gains in quality-adjusted life-years with TPF [13].

When IC is the chosen treatment, TPF is now the widely accepted gold-standard (Table 1). Immune checkpoint inhibitors are currently tested in combination with IC and might change the neoadjuvant paradigm in HNSCC (Table 2).

## 3. Attempts to Improve Induction Chemotherapy and Sequential Delivery

Despite all of this, toxicity with TPF can be a very important issue. Toxic death rates were reported as being between 2% and 7% in the different trials. Patients have to be treated and followed up by experienced oncologists. Multiple trials have tried to develop a less toxic and/or more efficient scheme of IC.

A retrospective analysis of 48 frail patients treated with modified TPF (docetaxel and cisplatin at 40 mg/m^2^ each on day 1, leucovorin 400 mg/m^2^, followed by a bolus of 5-FU at 400 mg/m^2^ and then 1000 mg/m^2^/day, days 1–2, every 2 weeks) in three French institutions [14] suggested similar efficacy with a response rate of 83%, with a considerably better tolerability compared with standard TPF. A randomized study in fit patients will compare this scheme and standard TPF. 

Other studies also tried to replace 5-FU with cetuximab, TPE (taxane, platinum, and cetuximab). The results of these small studies have been encouraging [15,16,17,18,19]. For example, a multicenter phase II study enrolled 92 patients with LA HNSCC to receive three cycles of docetaxel and cisplatin with or without cetuximab (TP and TPE) as induction chemotherapy. Patients in the TPE arm received CCR with cetuximab and cisplatin, whereas patients in the TP arm received cisplatin alone. In intention-to-treat analysis, the 3-year OS was not increased by cetuximab (88% vs. 74%, *p* = 0.31), possibly because cetuximab was responsible for a diminished treatment completion (67% vs. 77%). In per protocol analysis (IC + CCR completed, 67% in the TPE arm vs. 77%), the 3-year OS was significantly higher with cetuximab (94% vs. 73%, *p* = 0.045) [16].

Intensifying TPF has proven difficult, necessitating either a reduction in the dose of TPF or the removal of 5-FU [20,21]. A phase II trial of 50 patients added weekly cetuximab to TPF (C-TPF) for four cycles. Response rate was similar to TPF at 86% (95% CI: 73–94) but toxicity was highly increased with febrile neutropenia (24%), grade 3–4 diarrhea (20%) and grade 3–4 mucositis (14%) [21].

Another way to enhance efficacy might be by selecting patients earlier. A phase II trial tested a split-dose TPF (3-week cycle of docetaxel 30 mg/m^2^, cisplatin 40 mg/m^2^ and fluorouracil 2000 mg/m^2^/24 h at days 1 and 8) [22]. Responders after 1 cycle (radiological response >30%, 70% of patients), received 2 more cycles before surgery and CCRT. In non-responders, surgery was performed immediately before CCRT. All the patients benefited from surgery and major radiotherapy protocol deviations did not occur. As expected, 2-year PFS and OS were higher in responders (88.5% vs. 60.6% and 97.3% vs. 73.7% respectively). 

Another issue is that IC can compromise the delivery of the following RT/CCRT. To date, there is no standard approach for RT after IC in terms of whether it should be administered alone, with cisplatin, or with cetuximab.

A first phase II study, TREMPLIN, attempted to answer this and compared RT (70 Gy) with concurrent cisplatin q3w or concurrent cetuximab after three TPF cycles for organ preservation (stage III or IV larynx/hypopharynx) [23]. The primary endpoint was larynx preservation at 3 months: no significant difference (95% and 93%, respectively). The same result was found for OS at 18 months (92% and 89%, respectively). There were more local failures in the cetuximab group. RT with cisplatin was more toxic as only 42% of patients received the three planned cycles, whereas 71% of the patients in the cetuximab group received the seven planned injections.

In another phase III trial [24], 519 patients with inoperable stage III/IV HNSCC received TPF and if tolerated (and no progression) were randomized (407) to receive potentiation by either cisplatin or cetuximab. In an intention-to-treat analysis, the median OS was 42.2 months (95% CI: 33.7–52.4). This result underlines the efficacy of induction. Because of the excellent results of IC, this trial did not reach sufficient events and only interim data were presented. There was a non-significant tendency for cisplatin: 63.6 months (95% CI: 43.6–77.5) versus 47.1 months (95% CI: 33.7–NA) for OS (*p* = 1.17) and 37 months (95% CI: 23–62.9) versus 20.7 months (95% CI: 15.3–31.1) for PFS (*p* = 1.20). More time is needed to see if this trend in favor of cisplatin is confirmed and becomes significant.

## 4. Results and Controversies of Induction Chemotherapy for Unresectable Disease: Should it be Used for High-Risk Patients?

To date, the benefit of induction chemotherapy in clinical practice compared with the present standard CCRT remains controversial in inoperable/unresectable disease (Table 3). Five phase III trials compared the two approaches, but none allowed a definitive conclusion due to deficiencies in trial design, execution or insufficient patient accrual (Table 4).

A Spanish study by Hitt et al. randomized 439 patients with unresectable disease into three groups: exclusive chemoradiation (with three cycles of cisplatin) or preceded by induction PF or TPF [25]. After induction, it was difficult to proceed to chemoradiation as 47/155 in the TPF group and 42/156 in the PF group did not receive the planned treatment. In an intention-to-treat analysis, there was no significant difference in PFS (14.6, 14.3, and 13.8 months for TPF, PF, and CT/RT) or in OS (27, 27.2, and 26.6 months, respectively), but when analysis was carried out per-protocol, PFS tends to be superior in favor of induction: 20.4, 18.8, and 13.3 months, respectively, for TPF, PF, and CTRT (*p* = 0.083). Direct comparison between the TPF group and CT/RT for OS shows a significant benefit per-protocol in favor of induction (HR = 0.719; 95% CI: 0.526–0.983; *p* = 0.03). Of course, a selection bias of patients with a better prognosis cannot be excluded with this type of analysis but it might be that the sequential approach might offer better results for patients that can receive the entire course of therapy. Precise clinical and biological predictive markers are needed in order to determine which patients can benefit the most and also tolerate the whole sequential treatment.

Moreover, an American phase III trial evaluated the same question in 2013 but with major methodological flaws: 145 inoperable patients included for the 300 planned, and a weak survival hypothesis in the control arm with 55% at 3 years (and a hope of 70% with IC) [26]. The control arm received accelerated RT with boost and weekly cisplatin. After three TPF cycles, patients received standard RT with weekly carboplatin (in case of good response to IC) or accelerated RT with boost potentiated by docetaxel in case of a poor response. The 3-year OS was 78% (considerably beyond the hypothetical 55%) in the control group and 73% in the experimental group (HR = 1.05; 95% CI: 0.59–2.03; *p* = 0.77). 

The French GORTEC 2007-02 phase III trial randomized 370 patients to receive either concomitant CT/RT (carboplatin 70 mg/m^2^ day 1 and 5-FU 600 mg/m^2^/day for 4 days q3w) or IC with three TPF, followed by concomitant RT/cetuximab in case of response or stable disease [27]. The patients included had very advanced HNSCC (T2–T4, N2b–N3). Compliance to RT did not differ between the two arms. The response rate with IC was deceiving at 44.5% and 7% of toxic deaths were deplored during IC; there was no difference in PFS (median 11.5 vs. 12.5 months; HR = 0.95, *p* = 0.74) or OS (24.6 vs. 22.8 months; HR = 1.10; *p* = 0.48). It is note-worthy that there was a significant difference in distant metastases-free survival (HR = 0.62; *p* = 0.03). 

Ghi et al. published a phase II/II trial that randomized 414 mixed population of patients with stage III–IV low surgically curable or functionally inoperable HNSCC of the oral cavity, oropharynx, and hypopharynx to one of four treatments: concomitant chemoradiation (by cisplatin/5-FU × 2), RT with cetuximab, three cycles of TPF, followed by the same CRT or three cycles of TPF, followed by cetuximab/RT [28]. For analysis, data of the two IC arms and of the two exclusive CCRT were pooled. Results are significantly in favor of IC: Complete response was 43.5% in induction and 28% in the concomitant arm (*p* = 0.002) and the median PFS was 29.7 months versus 18.5, with a 3-year PFS of 46.8 versus 36.7% (HR = 0.73; 95% CI: 0.57–0.94; *p* = 0.015), respectively. The median OS was 53.7 versus 30.3 months, with a 3-year OS of 57.6 versus 45.7% (HR = 0.72; 95% CI: 0.55–0.96; *p* = 0.025), respectively. It seems that induction TPF, followed by CT/RT or cetuximab/RT significantly improved PFS and OS without compromising compliance to the concomitant treatments compared with CT/RT or cetuximab/RT alone. As there are doubts on the equivalence between cetuximab and cisplatin as RT sensitizing agents, no definitive conclusion can be drawn, especially as the subgroup analysis with only cisplatin as potentiation showed a non-significant trend (this analysis was not prespecified and the statistical power was probably too weak). Interpretation may also be confounded by the addition of cetuximab/RT only in the phase III portion.

Moreover, the other American trial, DECIDE, compared CCRT (docetaxel, fluorouracil, and hydroxyurea plus RT 0.15 Gy twice per day every other week) with two cycles of TPF, followed by the same CCRT in patients with N2–N3 disease [29]. Although 400 patients were initially planned, only 285 were included; thus, the statistical power was diminished. There was a trend in improved PFS and lower incidence of HNSCC-related death with TPF. Plus, patients with N2c/N3 disease had an improvement in OS with IC (*p* = 0.19). In a retrospective analysis, OS was 14 months with CCRT, while not reaching in the TPF arm [30]. Despite the bias that can be present in a retrospective study, this data is interesting to consider for these patients with high risk of distant failure (DF).

Indeed, these patients with large nodal disease, multiple involved nodes or low nodes seem to gain some benefit with IC followed by CCRT as can be seen in the DECIDE and the Spanish trial [25,29] and also in the French GORTEC trial where patients have a lower DF rate with the sequential treatment [27].

Urgently needed are predictive biomarkers that can identify patients that might benefit from the reduction of the risk of future distant metastases by the sequential chemotherapy approach. It seems for example that patients with present versus absent lower neck nodal involvement have a significantly lower 5-year distant metastasis-free survival rate (34.3% versus 55.2%, *p* = 0.008) as shown decades ago [31]. This numbers might be different today as therapeutics (chemotherapy, radiotherapy) have evolved. Other potential predictive biomarkers are being evaluated, like p53 or GDF15 [32,33].

## 5. Gold Standard for Laryngeal Preservation

The only widely accepted setting for IC is for laryngeal preservation in patients with resectable laryngeal or hypopharyngeal LA HNSCC. 

In 1991, PF IC followed by radiotherapy lead to a 31% complete response rate and 54% partial response rate in a phase III trial of 332 patients with previously untreated laryngeal LA SCCHN [34]. Survival rate with PF was similar to total laryngectomy (2-year survival of 68% in both groups, *p* = 0.9846), with more frequent local recurrences and fewer distant metastases.

A more recent trial with the same approach confirmed the results with PF [35]. The OS with a functional larynx was 45% and up to 36% at 3 and 5 years, respectively.

IC with TPF has shown even more efficacy in larynx preservation than PF as shown in the GORTEC 200-2001 trial [36]. This study compared induction by PF with TPF in 213 patients for organ preservation (stage III or IV larynx and hypopharynx) and confirmed that TPF increased larynx preservation and larynx dysfunction-free survival with possibly less toxicity. The 5- and 10-year larynx dysfunction-free survival rates were 74.0% (95% CI: 0.64–0.82) versus 58.1% (95% CI: 0.47–0.68) and 70.3% (95% CI: 0.58–0.8) versus 46.5% (95% CI: 0.31–0.63, *p* =0.01) in the TPF versus the PF arm, respectively. OS, disease-free survival, and locoregional control rates were not different, but TPF led to significantly fewer grade 3–4 late toxicities of the larynx (9.3% vs. 17.1%, *p* = 0.038).

RTOG 91-11 is the only study that compared sequential chemotherapy (PF) and radiation with CCRT and RT alone in patients with stage III or IV glottic or supraglottic cancer [37]. Concomitant cisplatin/RT significantly improved the larynx preservation rate over induction PF, followed by RT (HR = 0.58; 95% CI: 0.37–0.89; *p* = 0.005) and over RT alone (*p* < 0.001). However, the 10-year update indicated a significant improvement in laryngectomy-free survival and a trend in improved OS with sequential chemotherapy over concurrent chemoradiotherapy as well as a significantly greater number of non–treatment-related and non–disease-related deaths in the concurrent chemoradiotherapy arm.

All of these data show that sequential chemotherapy with TPF is the best long term option for laryngeal preservation. A definitive answer might come with the French ongoing SALTORL trial.

## 6. A Future Role in Treatment De-Escalation for HPV?

The prognosis of HPV-linked tumors is now established as being better than non-HPV tumors and better responses to treatment [38]. Multiple trials are evaluating the role of IC in treatment de-escalation.

In prospective, phase II, single-center trial, investigators classified patients as at low and high risk based on the American Joint Committee on Cancer staging system [39]. IC consisted three cycles of carboplatin and nanoparticle albumin-bound (nab)-paclitaxel. Low-risk patients who had more than 50% reduction in tumor volume received radiation therapy alone to 50 Gy, those with an intermediate response (30–50% reduction in volume) received low-dose chemoradiation to 45 Gy, and those who did not respond favorably received standard-dose chemoradiation to 75 Gy. High-risk patients who had more than 50% reduction in tumor volume received low-dose chemoradiation to 45 Gy, and in case of less than 50% response received standard-dose chemoradiation to 75 Gy. RT was delivered over 5 weeks in 2-Gy fractions daily. In the CCRT arms, treatment consisted of paclitaxel, fluorouracil, and hydroxyurea systemic therapy with 1.5-Gy radiation twice daily every other week. 82% of the patients were subsequently de-escalated. The response rate from induction therapy was 88.5%, 2-year PFS was 91% in low-risk patients and 94% in high-risk patients. Findings also demonstrated clear improvements in both acute and chronic toxicities with the de-escalation approach. Another example of de-escalation is the ongoing Quarterback trial, which will include 365 patients [40]. Those who respond to induction chemotherapy will be randomized to experimental treatment with reduced dose RT (56 Gy) and carboplatin versus standard treatment with RT (70 Gy) and carboplatin.

IC might have an interesting role in treatment deintensification in patients with favorable prognoses in the nearby future.

All these results are to be interpreted with caution. A recent trial showed there was a significant difference in the 2-year overall survival between cisplatin and cetuximab (97.5% vs. 89.4% respectively, *p* = 0.001, HR = 4.99, 95% CI: 1.70–14.67) and in 2-year recurrence rate (6.0% vs. 16.1% respectively, *p* = 0.0007, HR = 3.39, 95% CI: 1.61–7.19) in favor of cisplatin for HPV + OPC with exclusive CCRT [41].

## 7. Conclusions

IC has now been used for almost 30 years and still no clear guidelines exist concerning its use in LA HNSCC. TPF is clearly the gold standard when the sequential approach is considered and for laryngeal preservation. Multiple schemes were tested in order to reduce toxicity of TPF while maintaining efficacy but none has yet succeeded in a phase III trial. The role of IC in inoperable/unresectable disease is unclear compared to the standard CCRT and is probably of interest in high-risk patients. The sequential treatment sounds also interesting for treatment de-escalation in patients with good prognoses.

## Figures and Tables

**Table 1:** The two trials that made TPF (docetaxel, cisplatin, and fluorouracil) the standard of care. T: radiotherapy; PFS: progression-free survival; OS. overall survival. PF: cisplatin, and fluorouracil

Trial	Trial Scheme	Population	Results
TAX 324 [10]	TPF → carboplatin + RT vs. PF → carboplatin + RT	Inoperable and borderline resectable disease	OS significantly better with TPF
TAX 323/EORTC 24,971 [11]	TPF → RT vs. PF → RT	Inoperable disease	PFS and OS significantly better with TPF

**Table 2:** Ongoing trials of induction chemotherapy in association with immunotherapy.

TRIAL	Phase	Patients	Inclusion Criteria	Experimental Treatment
Pich (NCT03114280)	I/II	55	Stage III–V oral cavity, oropharynx and hypopharynx)	3 cycles of TPF + Pembrolizumab
NCT03342911	II	37	Stage III–IV oral cavity, oropharynx and hypopharynx)	3 cycles of carboplatin + paclitaxel + Nivolumab
Medinduction (NCT02997332)	I	36	Stage III–IV oral cavity, oropharynx and hypopharynx)	3 cycles of TPF + Durvalumab

**Table 3:** Potential advantages and disadvantages of induction chemotherapy (IC) compared to concurrent chemoradiotherapy (CCRT).

Advantages	Disadvantages
More beneficial to high risk patients	More toxic
More proven in laryngeal preservation	No proven survival benefit compared to CCRT
Useful for treatment de-escalation	

**Table 4:** Direct comparisons between Induction chemotherapy and concomitant chemoradiotherapy in unresectable disease HR: hazard ratio.

Trial	Trial Scheme	Population	Results
Hitt et al. [25]	TPF → CCRT (three cycles of cisplatin 100 mg/m^2^) vs. PF → CCRT vs. CCRT alone	Inoperable disease	No difference in OS or PFS in either arm
PARADIGM trial [26]	TPF → CCRT (with carboplatin or docetaxel) vs. CCRT (with two cycles of cisplatin 100 mg/m^2^)	High risk HNSCC: -stage III or IV -N2 or N3	No significant difference in OS (HR, 1.09; 95% CI 0.59–2.03)
GORTEC 2007-02 trial [27]	TPF → CCRT (with cetuximab) vs. CCRT (three cycles of carboplatin + fluorouracil)	High risk HNSCC: -stage III or IV -N2B/C and N3	-No statistically significant differences in PFS and OS (HR, 1.10; 95% CI 0.84–1.45) -Higher distant free metastasis survival in TPF arm (HR, 0.62; 95% CI 0.40–0.95)
Ghi et al. [28]	TPF → CCRT (with cisplatin of cetuximab) vs. CCRT alone (with cisplatin of cetuximab)	Stage III and IV HNSCC	OS higher with TPF than without
DeCIDE trial [29]	TPF → CCRT (with fluorouracil, docetaxel and hydroxyurea) vs. Same CCRT	N2 and N3 HNSCC	No statistically significant difference in OS (HR, 0.91; 95% CI, 0.59–1.41) Strong trend in OS for high risk tumors (N2c and N3)
